# Autologous Nonvascularized Fibula Graft and Locking Compression Plating for Failed Fixation of Humeral Shaft With Atrophic Gap Nonunion

**DOI:** 10.7759/cureus.24293

**Published:** 2022-04-19

**Authors:** Karan Shetty, Naga Cheppalli, Deepak Kaki

**Affiliations:** 1 Orthopaedics, Sapthagiri Institute of Medical Science and Research Centre, Bengaluru, IND; 2 Orthopaedics, University of New Mexico School of Medicine, Albuquerque, USA; 3 Orthopaedics, Balaji Institute of Surgery, Research and Rehabilitation for the Disabled (BIRRD) Hospital, Tirupati, IND

**Keywords:** gap nonunion, humeral fracture, nonunion, locking compression plating, humerus, autologous fibula graft

## Abstract

Introduction

The surgical treatment of humeral shaft atrophic, gap nonunion following failed surgical fixation is challenging. We intended to evaluate the surgical outcome of failed fixation of humeral shaft atrophic, gap nonunions using locking compression plate (LCP) and autologous nonvascularized fibular graft (ANVFG) and autologous iliac crest bone graft (AICBG).

Methods

Through our database search between 2015 and 2018, we identified 12 patients with humeral shaft atrophic, gap nonunions with failed surgical fixation underwent open reduction and internal fixation using LCP with autologous fibula graft and iliac crest cancellous bone graft.

Results

We have followed all twelve patients for a minimum period of 24 months. All patients had radiological and clinical union with a mean time to union of 17 weeks. In one case superficial surgical site infection was noted and successfully treated with intravenous antibiotics, and in another, transient peroneal nerve palsy was identified and resolved in six months.

Conclusion

LCP with ANVFG and AICBG is a reliable option for “complex” diaphyseal humerus atrophic and gap non unions, especially with significant bone loss. This construct provides mechanical stability and supports biological healing in these complex fractures.

## Introduction

Nonunion of humeral shaft fractures after failed osteosynthesis can be challenging to treat, especially in the presence of disuse osteoporosis, osteolysis secondary to implant loosening, or associated with significant bone loss and gap. Multiple surgical options have been described in the literature including open reduction and internal fixation with dynamic compression plate (DCP), locking compression plate (LCP) interlocking nail (ILN), and external fixator [1]. Autologous nonvascularized fibular graft (ANVFG) has been used in combination with plating [2-4]. This construct improves biomechanical strength and provides enhanced biology for gap and atrophic nonunions in long bones. The purpose of this study is to assess the outcome of surgically treated “complex” humerus shaft nonunion (failed primary osteosynthesis, with atrophic and gap nonunion) using a combination of LCP, ANVFG with autologous iliac crest bone graft (AICBG).

## Materials and methods

Institution Ethical Committee approval was obtained for this retrospective study. Patients' informed consent is taken at the time of collection of final follow-up and collection of DASH scores. Patients were identified by searching through our institute's register from 2015 to 2018 for operated cases for humeral fracture nonunion fixation. We also searched through the surgeon's operative logbook to avoid any missing data. (No electronic records are available at our institution). We defined “complex humerus nonunion” as an established nonunion (patient presented clinically with painless abnormal mobility, with radiological signs suggesting the fracture failed to unite) of the humeral shaft after failed osteosynthesis and associated with the significant gap (minimum 5 mm), and osteolysis at the screw and bone interface with atrophic fracture end. We included only “complex” humeral nonunion for this study.

We identified 12 patients and the senior surgeon determined that all these patients needed additional mechanical stabilization in the form of intramedullary cortical strut in addition to locking compression plating (LCP). As the bone bank is not available and affordable in our region, the senior surgeon elected to use ipsilateral fibula as a nonvascularized cortical strut graft to provide additional mechanical stability and autologous iliac crest cancellous graft for osteoinduction at the fracture site along with LCP. Closure of the gap is performed whenever possible. All 12 patients were followed up regularly clinically and radiologically for a minimum period of two years. There was no loss of follow-up. We excluded infected nonunion, and aseptic nonunion after conservative management for this study.

Surgical technique

**Figure 1:**
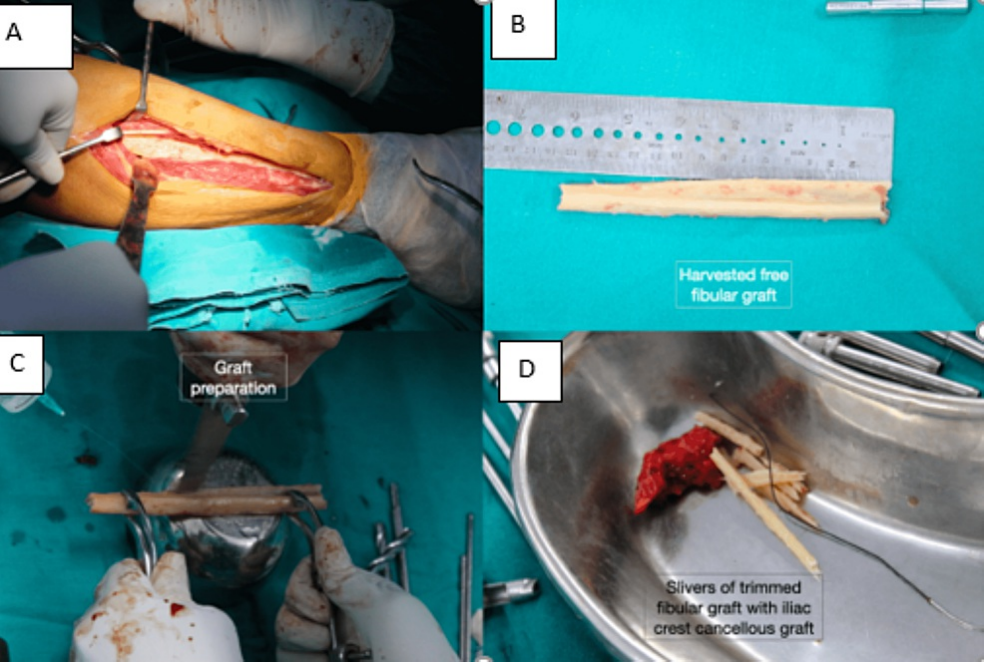
Technique of fibular graft harvesting and preparation. (A) Exposure of fibula. (B) Harvested fibular graft. (C) Preparation of fibular graft. (D) Slivers of trimmed fibular graft along with cancellous Iliac crest autograft used at the nonunion site.

All cases were operated on under general anesthesia. The surgical approach and hence patient position were based on the previous surgical approach. One team of surgeons harvested fibular graft, while the other team removed humeral hardware and prepared the nonunion site. Fibular graft harvesting was performed under a tourniquet. The ipsilateral knee was flexed and the fibula was approached via a lateral approach. The graft was to be harvested in the central third of the fibula, ensuring at least 7 cm of the fibula is preserved proximally and distally to avoid peroneal nerve injury and ankle/knee instability. The length of the harvested graft ranged from 9-15 cm. Multiple drill holes were made at desired levels proximally and distally before completing the osteotomy with osteotomes (Figures 1A-1D).

Humerus was exposed following the previous surgical approach used for primary fixation. Previous implants were removed, soft-tissue scarring and intervening fibrous tissue were excised and the bone was debrided. Radial nerve neurolysis was done whenever necessary. Fracture ends were freshened. With serial reaming using rigid reamers, the medullary canal was recanalized in both fragments. The fibular graft was prepared such that its diameter was snuggly fitting the endosteal diameter of the reamed humerus. The center of the graft was marked and placed as an intramedullary strut, ensuring the center was at the level of the fracture. The average length of graft harvested was 11.66 cm, and the average length of graft used was 10.58 cm. Slivers of the harvested fibular strut obtained during graft preparation were included with iliac crest graft autograft at nonunion. End-to-end compression was achieved in nine cases with an articulated tensioner and using dynamic holes and nonlocking screws on either fragment, while three cases with severe preoperative shortening required structural tricortical iliac crest autograft to bridge the gap across the ends. Iliac crest cancellous bone grafting was done at the graft-humerus interface in all cases, to augment chances for the union. Fixation was done with a 10-holed 4.5 mm LCP, contoured to match the cortical surface. In all the cases, the proximal fragment was stabilized with eight cortices, however, based on the available bone distally four to eight humeral cortices were fixed with locking screws. Emphasis was placed on ensuring at least two screws in each fragment passing through fibular graft in addition to humeral cortices.

Postoperative follow-up

Postoperatively, the limb was immobilized in a sling for four weeks. Four weeks post-operatively, active-assisted shoulder and elbow exercises were started. Patients were followed up once every four weeks until the radiological union was seen and then at three months intervals. The minimum follow-up was 24 months. Functional assessment was done by using the disabilities of the arm, shoulder, and hand (DASH) score both preoperatively and postoperatively at the final follow-up.

## Results

**Figure 2:**
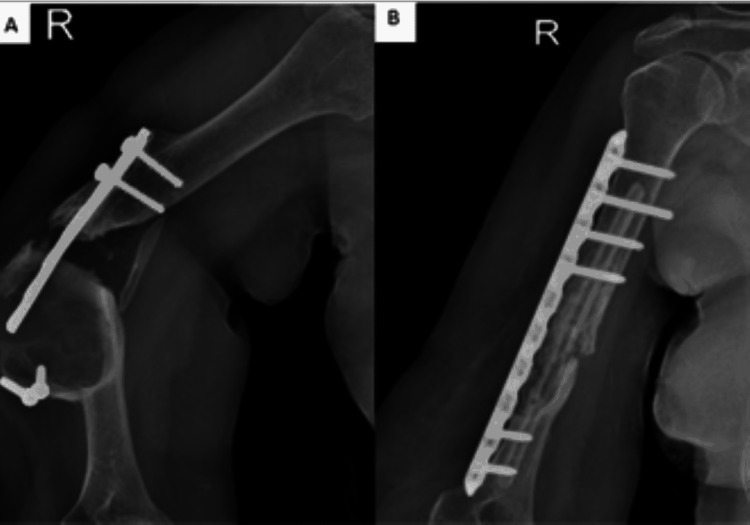
(A) Radiographs demonstrating non-union of the humerus after failed osteosynthesis. (B) Postoperative radiograph demonstrating healed fracture with locking compression plate and fibular graft.

**Figure 3:**
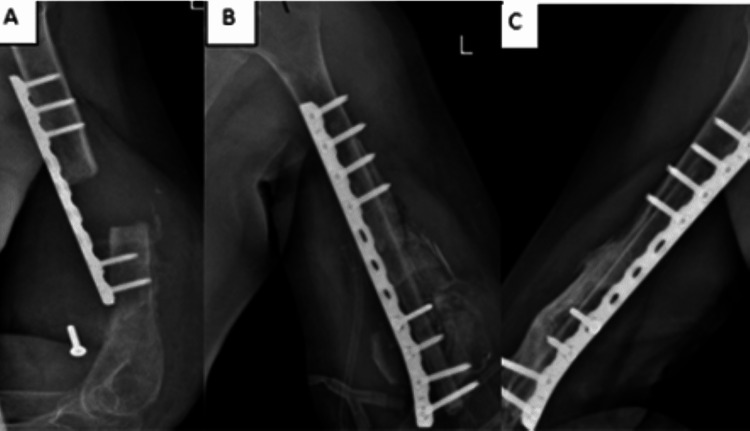
(A) Radiographs showing nonunion with failed osteosynthesis. (B) Immediate postoperative radiograph with LCP and fibular graft. (C) Postoperative image showing healed fracture. LCP: Locking Compression Plating

**Figure 4:**
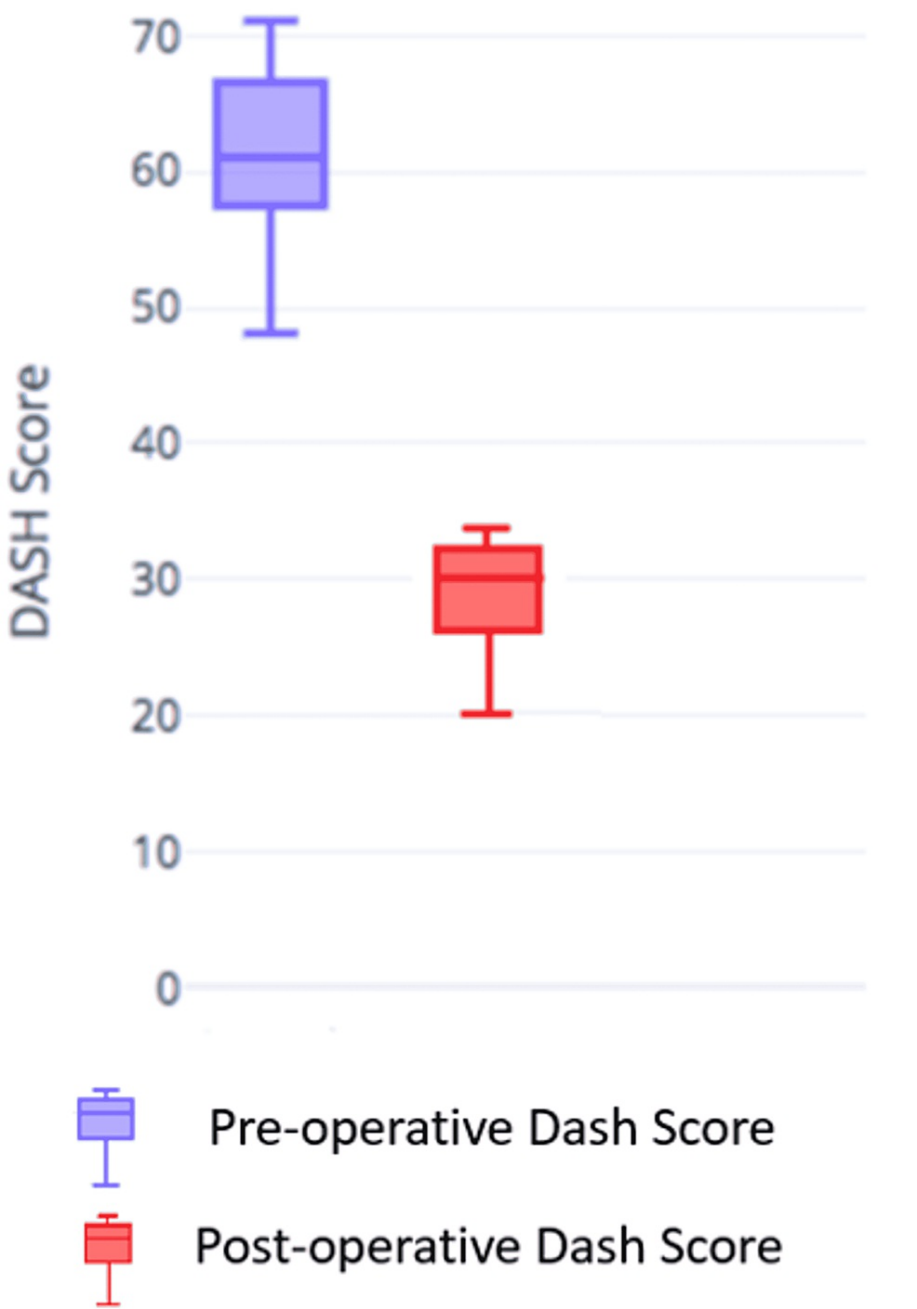
Graphical representation of preoperative and postoperative DASH score. DASH - disabilities of the arm, shoulder, and hand

Twelve patients were identified as fulfilling the criteria for “complex” humeral nonunion following failed primary osteosynthesis. The mean age of the study group was 50.7 (±6.92) years. Duration of nonunion, i.e., time since index surgery ranged from six months to three years. The bone gap ranged from 5 mm to 60 mm. Patients had undergone at least one to three prior surgeries. All the fractures were at the level of the middle third except one which was at the junction of the middle third and distal third. We excluded any patients with signs suggestive of infection. ESR and CRP values were checked for all patients to rule out subclinical infection. When in doubt, intraoperative tissue was sent for cultures and treated with antibiotics until the culture results are back (2/12 patients). Each patient was treated with open reduction and internal fixation using 10 holed 4.5 mm LCP with Autologous fibular graft and iliac crest cancellous bone graft in all cases. All patients were followed up for a minimum period of two years. All patients had a clinical and radiological union. The radiological union was defined by the presence of fracture union in at least three of the four cortices in orthogonal x-ray views. No patient-reported pain at the fracture site. Meantime to radiological union was 17 weeks (range, 12-24 weeks). When clinically measured the average arm shortening was 8 mm (range, 5 to 20 mm) compared to the contralateral side. Shoulder abduction and flexion were found to be reduced by an average of 15 degrees each, postoperatively. However, preoperative flexion stiffness of the elbow resolved partially in all cases. Patient demographics, surgical indications, and details of the surgery with the outcome are presented (Table 1) and case examples of radiographs are presented (Figures 2A, 2B, 3A-3C). All patients returned to near normal pre-injury activity levels, with DASH scores improving from a preoperative average of 61.0 ± 6.7 (range, 48.1 - 68.2) to an average final follow-up DASH score of 28.8 ± 4.4 (range, 19.9-33.3), with a mean improvement of 32.23 (Figure 4). All patients were satisfied with the treatment with none having any wound complications, except one patient who developed a superficial surgical wound infection which resolved with debridement and intravenous antibiotics. One other patient developed transient peroneal nerve palsy which showed signs of recovery in seven weeks and completely resolved in six months. No graft site morbidity was noted at the final follow-up.

**Table 1:** Patient demographics,  surgical indications, and details  of the surgery with the outcome.

Sl No.	Age (yr)	Sex	Level	Type of non-union	Duration of non-union (months)	Prior surgeries	Risk factors	Fibular graft length (cm)	Implant used	Time for union (months)	Complications	Pre-op DASH score	Final follow-up DASH score	Improvement
1	58	M	Mid 1/3rd	Atrophic	6	1	Smoking, osteoporosis	12	LCP	3	nil	68.2	12.4	55.8
2	45	F	Mid 1/3rd	Atrophic	11	1	nil	12	LCP	5	nil	66.7	30.1	36.6
3	48	M	Mid 1/3rd	Atrophic	8	1	nil	9	LCP	4	nil	61.4	28.3	33.1
4	52	M	Mid 1/3rd	Comminuted& atrophic	20	1	Smoking	10	LCP	4	nil	55.8	31.8	24
5	55	F	Mid-distal 1/3rd junction	Atrophic	8	2	Diabetes mellitus	15	LCP	4	Surgical site infection	66.5	19.9	46.6
6	61	F	Mid 1/3rd	Comminuted atrophic	6	1	Diabetes, hypothyroidism	15	LCP	5	nil	52.1	29.6	22.5
7	37	M	Mid 1/3rd	Atrophic	24	1	nil	13	LCP	5	nil	48.1	21.7	26.4
8	53	F	Mid 1/3rd	Atrophic	10	1	Diabetes mellitus	10	LCP	4	nil	62.2	33.3	28.9
9	44	M	Mid 1/3rd	Atrophic	28	3	Smoking	12	LCP	4	nil	71.1	31.4	39.7
10	45	F	Mid 1/3rd	Atrophic	24	1	nil	12	LCP	4	nil	60.7	24.7	36
11	51	F	Mid 1/3rd	Atrophic	13	1	Hypertension	10	LCP	5	nil	59.2	30.1	29.1
12	60	F	Mid 1/3rd	Atrophic	12	1	nil	10	LCP	4	nil	60.1	32.0	28.1

## Discussion

Nonunion of the humeral shaft is likely to be related to the pattern of fracture, soft-tissue interposition, and quality of primary fixation [5]. Failure to unite after surgical treatment may be due to poor contact between the fracture ends, inadequate stabilization, devitalization of bone, osteopenia, and bone defects. Smoking, obesity, alcoholism, diabetes mellitus, and method of treatment may also be contributory factors [6]. We defined “complex humerus nonunion” as the established nonunion of the humeral shaft after failed osteosynthesis, and associated with significant gap nonunion (at least 5 mm), and osteolysis at the screw and bone interface. The patients included in this study had atrophic type gap nonunion, with failed osteosynthesis attempts from these aforementioned local and general factors. Local osteoporosis, mostly secondary to disuse, compounds the choice of surgical treatment in these patients. Often, these patients had implant breakage, metal debris, and metallosis, with scalloping around the screws and ballooning of the cortex. This poses a reconstructive challenge, and various options have been described for fracture fixation earlier in the literature.

Numerous surgical options (plating, nailing, external fixator) have been described to treat humeral nonunion with bone defects such as primary shortening, one stage cancellous bone graft, cancellous bone graft in two stages (after induced membrane), pedicle bone transfer (free border of the scapula, ninth rib), periosteal free flap transfer or bone morphogenetic protein [7-10].

We choose LCP as our treatment option for fracture stabilization as we are more familiar with this approach. In our hands, we believe that the ILN is not suitable for some distal fractures, violates rotator cuff integrity, and depends on an image intensifier and technician (adding to the cost of the procedure) [11]. Bone transport using distraction histogenesis with Ilizarov fixators or limb reconstruction system has a certain appeal, but cost, technically demanding nature of this technique, the commitment of time, and complications have limited its acceptance [12]. Dynamic compression plating has been described and high rates of the union have been claimed. It provides compression at the fracture site, with correction of axial malalignment. It can be applied with great success provided the bone quality is reasonable. However, most cases of implant failure have poor bone quality and screw purchase, where instead of DCP, LCP may be a reliable option [13].

LCPs are very promising, especially in the presence of nonunion and osteopenia by providing a rigid, angle-stable construct [14]. Osteopenia as a result of disuse, due to a generalized metabolic cause and previous implant stress shielding along with previous screw holes will interfere with the strength and purchase of the screws inserted subsequently during osteosynthesis. The concept of quadricortical plating through intramedullary fibular strut graft was first described by Wright et al. [15]. This improved mechanical environment may reduce the risk of fibrous nonunion or failed fixation that can occur as a result of excessive motion and osteoporotic bone. Iliac crest bone graft (ICBG) has no inherent mechanical strength to withstand forces until fracture union and also contributes to significant graft harvest site morbidity. In our case series, we elected to use fibular graft for all the cases to add additional mechanical strength to the construction. An autologous iliac crest graft is added additionally at the fracture site. No deliberate shortening is performed. The nonvascularized fibula is easy to harvest with minimal harvest site morbidity and does not need expertise with microsurgical skills. The cortical bone affords immediate structural continuity and stability at the fracture site post-fixation. The fibula functions as a triflanged nail and engages the host bone firmly thus making it the most suitable donor bone for the reconstruction of defects in a long bone [16]. Although it is believed that cortical bone grafts frequently fail as surrounding soft tissue cannot provide adequate vascularity for the incorporation of graft, we did not find any graft being resorbed or failing.

While compared to surgical options including allografts, bone transport with external fixators, induced membrane technique, and BMPs, autograft with fibula is a significantly more cost-effective procedure. Even though the superiority of autologous non vascularized autologous fibular graft cannot be proven over allogenous fibular graft [16] or vascularized fibular graft, we believe this is an excellent option, especially in countries where the bone banking system is not well developed and there is a dearth of specialists with microsurgical skills. Therefore, this technique with fibular autograft will have greater acceptance as a reliable procedure that can be performed at most centers without significant financial burden to the patient (in countries where health care cost is directly paid by the patients). A possible theoretical disadvantage of ANVFG includes disruption of both the periosteal and the endosteal blood supply. Donor site morbidity is another disadvantage, although the impairment is often minimal [17-19]. Free nonvascularized fibular grafts are known to be at risk for necrosis resulting in absorption and nonincorporation to the host bone. However, it has been reported that if the autologous bone graft is fixed to the recipient's bone immediately after it is retrieved, the graft will survive. Immediate fixation of the graft at the recipient site allows the cells in and over the graft to draw oxygenation and nutrition from the blood, thereby preserving their osteogenic properties [17]. Complications reported from this technique include stress supracondylar fracture, radial nerve palsy, iatrogenic splinter during implant removal, transient peroneal nerve injury, and adhesive capsulitis of the shoulder [20]. However, we did not incur these problems.

The results in our series with minimal complications favor this easy and reliable option. One patient developed a superficial wound infection which was controlled with debridement with implant retention and parenteral antibiotics. Another patient developed transient peroneal nerve palsy which recovered completely in six months. 

This study has significant limitations. This is a retrospective case series with a limited number of patients (12) performed at a single center with limited resources (nonavailability of allograft and bone morphogenic protein), by a single surgeon. 

## Conclusions

Based on our experience, we conclude that autologous nonvascularized fibular strut graft with iliac crest cancellous autograft and fixation with LCP is a reliable option for treating “complex” diaphyseal humerus nonunion with significant bone loss and osteopenia. The addition of free fibular graft provided additional mechanical stability in osteoporotic bone, while AICBG improved the biologic milieu of repaired fracture. This technique resulted in a mean improvement of DASH score by 32.33, all patients returned to pre-injury level and resulted in 100% radiological union despite multiple previous failed fixations. This procedure is reproducible and can be performed with minimal morbidity. This procedure can be one of the treatment options in less resourceful regions (where the bone bank/allograft/BMP is not available).

## References

[1] Peters RM, Claessen FM, Doornberg JN, Kolovich GP, Diercks RL, van den Bekerom MP (2015). Union rate after operative treatment of humeral shaft nonunion--A systematic review. Injury.

[2] Gopisankar G, Justin AS, Nithyananth M, Cherian VM, Lee VN (2011). Non-vascularised fibular graft as an intramedullary strut for infected non-union of the humerus. J Orthop Surg (Hong Kong).

[3] Padhye KP, Kulkarni VS, Kulkarni GS (2013). Plating, nailing, external fixation, and fibular strut grafting for non-union of humeral shaft fractures. J Orthop Surg (Hong Kong).

[4] Farouk O, El-Sherif E, Mostafa K, Khalil A (2008). Intramedullary fibular graft and quadricortical plate fixation in atrophic non-union of the osteoporotic humerus. Inj Extra.

[5] Castell FB, Garcia FB, Berry EM, Perell EB, Sanchez-Alepuz E, Gabarda R (2004). Nonunion of the humeral shaft: long lateral butterfly fracture a nonunion predictive pattern?. Clin Orthop.

[6] Pascarella R, Ponziani L, Ferri M, Ercolani C, Zinghi GF (2000). Aseptic nonunion of the humeral shaft. Chir Organi Mov.

[7] Dimitriou R, Dahabreh Z, Katsoulis E, Matthews SJ, Branfoot T, Giannoudis PV (2005). Application of recombinant BMP-7 on persistent upper and lower limb non-unions. Injury.

[8] Patel VR, Menon DK, Pool RD, Simonis RB (2000). Nonunion of the humerus after failure of surgical treatment. Management using the Ilizarov circular fixator. J Bone Joint Surg Br.

[9] Barquet A, Fernandez A, Luvizio J, Masliah R (1989). A combined therapeutic protocol for aseptic nonunion of the humeral shaft: a report of 25 cases. J Trauma.

[10] Pinsolle V, Tessier R, Casoli V, Martin D, Baudet J (2007). The pedicled vascularised scapular bone flap for proximal humerus reconstruction and short humeral stump lengthening. J Plast Reconstr Aesthet Surg.

[11] Singh AK, Arun GR, Narsaria N, Srivastava A (2014). Treatment of non-union of humerus diaphyseal fractures: a prospective study comparing interlocking nail and locking compression plate. Arch Orthop Trauma Surg.

[12] Tomić S, Bumbasirević M, Lesić A, Mitković M, Atkinson HD (2007). Ilizarov frame fixation without bone graft for atrophic humeral shaft nonunion: 28 patients with a minimum 2-year follow-up. J Orthop Trauma.

[13] Hierholzer C, Sama D, Toro JB, Peterson M, Helfet DL (2006). Plate fixation of ununited humeral shaft fractures: effect of type of bone graft on healing. J Bone Joint Surg Am.

[14] Babhulkar S, Babhulkar S, Vasudev A (2017). Recalcitrant aseptic atrophic non-union of the shaft of the humerus after failure of surgical treatment: management by excision of non-union, bone grafting and stabilization by LCP in different modes. Injury.

[15] Wright TW (1997). Treatment of humeral diaphyseal nonunions in patients with severely compromised bone. J South Orthop Assoc.

[16] Willis MP, Brooks JP, Badman BL, Gaines RJ, Mighell MA, Sanders RW (2013). Treatment of atrophic diaphyseal humeral nonunions with compressive locked plating and augmented with an intramedullary strut allograft. J Orthop Trauma.

[17] Yadav SS (2018). The use of a free fibular strut as a "biological intramedullary nail" for the treatment of complex nonunion of long bones. JB JS Open Access.

[18] Kashayi-Chowdojirao S, Vallurupalli A, Chilakamarri VK, Patnala C, Chodavarapu LM, Kancherla NR, Khazi Syed AH (2017). Role of autologous non-vascularised intramedullary fibular strut graft in humeral shaft nonunions following failed plating. J Clin Orthop Trauma.

[19] Pollon T, Reina N, Delclaux S, Bonnevialle P, Mansat P, Bonnevialle N (2017). Persistent non-union of the humeral shaft treated by plating and autologous bone grafting. Int Orthop.

[20] Vidyadhara S, Vamsi K, Rao SK, Gnanadoss JJ, Pandian S (2009). Use of intramedullary fibular strut graft: a novel adjunct to plating in the treatment of osteoporotic humeral shaft nonunion. Int Orthop.

